# Prevalence of 25-OH-Vitamin D and Calcium Deficiency in Adolescent Idiopathic Scoliosis

**DOI:** 10.25122/jml-2020-0101

**Published:** 2020

**Authors:** Alexandru Herdea, Adham Charkaoui, Alexandru Ulici

**Affiliations:** 1. Department of Pediatric Orthopedics, “Grigore Alexandrescu” Emergency Hospital for Children, Bucharest, Romania; 2. “Carol Davila” University of Medicine and Pharmacy, Bucharest, Romania; 3. Department of Pediatric Surgery, Municipal Emergency Hospital of Moinesti, Moinesti, Romania; 4. Faculty of Medicine and Pharmacy, “Dunarea de Jos” University of Galati, Galati, Romania

**Keywords:** Adolescent idiopathic scoliosis, vitamin D, calcium

## Abstract

Several etiologies have been proposed as a basis and evolution theory for the development of adolescent idiopathic scoliosis, but limited data were published until now that link vitamin D and calcium deficiency to this condition. The present study aims to evaluate the relationship between 25-OH-Vitamin D, total calcium, and the following data: Cobb angle, age, and patient sex. The seasonal variation for vitamin D will also be taken into consideration. A total of 101 patients with a mean age of 11.61 ± 2.33 years had vitamin D and calcium levels tested. The mean Cobb angle was 26.21o ± 12.37. The level of vitamin D was, on average, 24 ng/mL ± 9.64. Calcium values were within the normal range, with an average of 9.82 mg/dL ± 0.42. The male group showed lower levels of vitamin D compared to the female group (19.6 vs. 25.45 ng/mL) (p = 0.02). Seasonal variations showed significant differences for vitamin D (p=.0001). Vitamin D level was positively correlated with the calcium level (p=0.01, r=0.973), but also with the patient’s age (p <0.001, r=0.158). The Cobb angle was negatively correlated with serum vitamin D levels (p<0.01, r=-0.472). Patients included in this study had low vitamin D levels, significant differences being observed between boys and girls, boys being more affected. The positive correlation between vitamin D and calcium, together with the negative correlation with the Cobb angle, is yet another proof that patients with idiopathic scoliosis should be investigated regularly for these pathologies.

## Introduction

Idiopathic scoliosis represents a complex deformity of the spine [1]. Even if it is postulated as an idiopathic disease, with an unknown cause, several etiologies have been proposed as a basis for the development of scoliosis [2].

Idiopathic scoliosis can affect between 0.47 and 5.2% of adolescents [3], but it is commonly accepted that it has a prevalence between 2 and 4%.

The genetic factor, namely a positive family history of scoliosis, leads to a 38% chance of further inheriting scoliosis, while the remaining 62% is attributed to independent factors [4]. Hormonal factors have been studied intensively over time in relation to the onset and evolution of scoliosis, such as melatonin [5,6], growth hormones [7], estrogen [8], calmodulin [9].

Some studies have shown that low bone density and osteopenia may manifest in patients with idiopathic scoliosis and may influence its appearance and development [10-12]. An optimal level of vitamin D and calcium contributes to good bone density, thus reducing the risk of bone fractures and also improving neuromuscular function [13, 14]. Osteoporosis and low vitamin D levels have been shown to be part of the patient’s profile with idiopathic scoliosis in a series of studies [15-18]. As the Cobb angle increases, calcium and Vitamin D levels decrease [17]. Recently, in a study, vitamin D and calcium were administered to patients with idiopathic scoliosis with positive results [18].

Given the important role that vitamin D and calcium play in the etiopathogenesis and evolution of idiopathic scoliosis, the present study aims to evaluate the relationship between 25-OH-Vitamin D, total calcium, and the following data: Cobb angle, age, and patient sex. The seasonal variation for vitamin D will also be taken into consideration.

## Material and Methods

### Study design and population

The study was done in an urban area at an emergency hospital for children in the Pediatric Orthopedic outpatient clinic between June 2017 – July 2019.

We chose to conduct a prospective, unblinded, non-interventional study for 101 patients diagnosed with adolescent idiopathic scoliosis, with a Cobb angle higher than 10o. The exclusion criteria were: scoliosis other than idiopathic (lower limb inequality, congenital malformations, hemivertebrae, muscle dystrophy), postural scoliosis, known metabolic or endocrine diseases, patients who previously had a fracture history. All procedures were in accordance with the ethical standards of the institutional research committee (Centre of Postgraduate Medical Education) and the 1964 Helsinki declaration and its later amendments or comparable ethical standards. This study was approved by the Ethics Committee of Grigore Alexandrescu Emergency Hospital for Children and written informed consent was obtained from the parents of all the participants.

## Study protocol

Patients who met the above-mentioned criteria were invited to undergo the following blood tests: 25-OH-Vitamin D and total calcium level. 25-OH-Vitamin D was considered normal with values between 30 ng/mL-100 ng/mL, insufficient between 20ng/mL-29ng/mL, or deficient when the values were below 20 ng/mL. Total calcium was considered normal with values between 8.80 mg/dL – 10.6 mg/dL.

Total calcium was analyzed in the laboratory by the spectrophotometric method on the AU680 biochemistry analyzer, developed on the AU640 Olympus platform. The chemiluminescent microparticle immunoassay (CMIA) method on the Abbott Architect analyzer was used in the laboratory to test Vitamin D (25-OH-Vitamin D) levels.

### Collection of data and statistical analysis

Demographic data, vitamin D, calcium, and Cobb angle were analyzed statistically. Sex was analyzed using the χ2 test. Seasonal effects were calculated using one-way ANOVA to determine whether there are any statistically significant differences between the moment of the year (spring, summer, autumn, winter) and the serum levels of calcium and Vitamin D. Pearson’s product-moment correlation was used to see the correlation between vitamin D level, age, and Cobb angle. Statistical significance was determined using a P value of less than 0.05 and a 95% confidence interval. Analyses were performed with the Statistical Package for the Social Sciences (SPSS) v.18.0 software.

## Results

One hundred one patients diagnosed with idiopathic scoliosis aged between 8 and 16 years met the inclusion criteria and were evaluated and followed in our institution between June 2017 and July 2019. The patients’ average age was 11.61 ± 2.33 years, with a 95% CI of 11.15 to 12.06, as shown in Table 1.

**Table 1:** Distribution of patients by age, level of vitamin D, calcium, and Cobb angle in the patient group.

	**Age**	**25-OH-vitamin D**	**Calcium**	**Cobb angle**
**Total**	101	101	101	101
**Average**	11.61	24	9.82	26.21
**SD**	2.33	9.64	0.42	12.37
**Median**	12	22	9.8	28
**Min**	8	9.3	9	11
**Max**	16	42	10.5	60
**95% CI**	[11.15. 12.06]	[22.12. 25.88]	[9.73. 9.90]	[23.79. 28.62]

The level of vitamin D in the group of patients was, on average, 24 ng/mL ± 9.64, with a 95% CI between 22.12 and 25.88. All patients had calcium values within the normal range, with an average of 9.82 mg/dL ± 0.42. The mean Cobb angle in the patient group was 26.21o ± 12.37, with a 95% CI between 23.79 and 28.62, the maximum being 60 degrees and the minimum 11 degrees.

The distribution of the 25-OH-vitamin D level divided by categories for the patient group is shown in Table 2. Almost three-quarters of the patients (72.27%) showed sub-normal values of vitamin D. A good part of the study group (40.59%) entered the category of patients with deficient serum vitamin D and 31.68% patients had a poor level.

**Table 2:** Overall visualization of the vitamin D level in the group of patients.

**Category**	**25-OH-vitamin D (ng/mL)**	**Number (%) of patients**
**Normal**	>30	28 (27.72%)
**Insufficient**	20-29	32 (31.68%)
**Deficient**	<20	41 (40.59%)

Analysis of the vitamin D level in correlation with sex showed a predominantly lower level for the male group (on average, 19.6 ng/mL) compared to the female group (on average, 25.45 ng/mL) being statistically significant (p = 0.02), as shown in Table 3.

**Table 3:** Comparison between the Vitamin D level and the patient’s gender.

**25-OH-Vitamin D**	**Female**	**Male**
**Total**	76	25
**Average**	25.45	19.6
**SD**	10.26	5.43
**Median**	22	19
**Min**	9.3	13
**Max**	42	30
P*	0.02	
**95% CI**	[23.14. 27.75]	[17.47. 21.72]

Note: *Chi-Square test.

Seasonal variations of Vitamin D are shown in Table 4, where significant differences are observed (p=.0001). Spring has the lowest average level of vitamin D (16.6 ng/mL on average).

**Table 4:** Comparison between Vitamin D levels and the season in which the patient was analyzed.

**25-OH-Vitamin D**	**Winter**	**Spring**	**Summer**	**Autumn**
**Total**	5	15	52	29
**Mean**	28	16.6	24.78	25.5
**SD**	5.12	5.23	8.72	9.19
P*	.000134			

Note: *One-way ANOVA.

In Table 5, statistical correlations were made between the 25-OH-Vitamin D level, calcium, patient age, and Cobb angle. Vitamin D level was positively correlated with calcium level (p=0.01, r=0.973), but also with the patient’s age (p <0.001, r=0.158). Cobb angle was negatively correlated with serum vitamin D levels (p<0.01, r=-0.472).

**Table 5:** Association between Vitamin D level, calcium level, patient age and Cobb angle.

**25-OH-Vitamin D**	**Calcium**	**Age**	**Cobb angle**
**r**	0.0973	0.158	-0.472
**P**	0.001	< .001	< .001

Note: *Pearson’s correlation test.

While using as a cut-off point of 30 degrees for the Cobb angle, it can be seen that the serum level of 25-OH-vitamin D decreases as the Cobb angle increases, as shown in Table 6 (p=0.06).

**Table 6:** Comparison between the Cobb angle and the Vitamin D level for a Cobb angle lower and higher than 30°.

**25-OH-Vitamin D**	**<30**° **Cobb angle**	**>30**° **Cobb angle**
**Total**	64	37
**Mean Vitamin D**	26.65	19.41
**SD**	8.81	9.29
P*	0.06	
**95% CI**	[24.49. 28.80]	[16.41. 22.40]

Note: *T-Test.

## Discussion

Children may suffer from insufficient vitamin D levels for various reasons, such as the cold season (winter) when there is less sun exposure, but also insufficient dietary intake of Vitamin D [20, 21]. Dietary intake of Vitamin D, sun exposure, and exercise levels were not quantified in the present study. All patients were asked if they use vitamin D, calcium, or other supplements, and the answer was negative in both cases.

The optimal vitamin D level, measured by 25-OH-Vitamin D, was proposed at a value of over 30 ng/ml, with an ideal value between 40 and 60 ng/ml. Deficiency is considered when the level is below 20 ng/ml, and between 20 and 29 ng/ml, the patient is considered to have an insufficient level [23, 24].

Some authors have shown that patients with adolescent idiopathic scoliosis have a lower level of vitamin D compared to a control group [17,25]. One of the limitations of the present study is the lack of comparison with an age-matched control group. However, a low level of vitamin D will impact bone mineral density and the development of deformities, such as idiopathic scoliosis of the adolescent [26, 27].

It is assumed that 25-OH-vitamin D is associated with the onset and development of idiopathic scoliosis of the adolescent. The results of the current study showed that the serum level of 25-OH-vitamin D was, on average, well below the benchmarks. Our results are in line with the results obtained by other authors [16, 28, 29], demonstrating that the level of 25-hydroxyvitamin D was lower in patients with idiopathic scoliosis compared with a healthy group of patients.

Calcium levels appear to be within normal limits in patients with idiopathic scoliosis [15-18]. The obtained values were within the reference range for the studied population. In a study by Batista et al., it was observed that not only calcium, but also the levels of phosphorus (phosphate) and parathyroid hormone (PTH) of the subjects were normal, both in the patients in the study group and the healthy patients in the control group [16].

The role of vitamin D is not limited only to the metabolic relationship between calcium, and vitamin D. Vitamin D can also influence cardiac function, autoimmune diseases, and infectious diseases [30, 31].

Seasonal variation can be an essential factor to consider when evaluating the role of vitamin D in scoliosis. In our study, there were statistically significant differences between groups, and all 101 patients were evaluated for seasonal effects. The lowest level was found during spring, averaging at 16.6 ng/mL, compared to the study by Balioglu et al., which noted that patients had the lowest level (14.41 ng/mL) during winter [17]. However, in our study, there were only five patients during the winter season, and a larger group of patients could bring other results. Studies in larger populations are needed to observe the variation in 25-OH-vitamin D levels observed between study groups and healthy controls group.

## Conclusions

The patients included in this study had low Vitamin D levels, significant differences being observed between boys and girls, boys being more affected. The positive correlation between Vitamin D and calcium, together with the negative correlation with the Cobb Angle, is yet another proof that patients with idiopathic scoliosis should be investigated regularly for these pathologies.

Vitamin D may be a much more significant player than has been considered so far in the onset and aggravation of idiopathic scoliosis. More extensive studies, in larger populations, should be conducted to observe if vitamin D and calcium administration may have the effect of slowing or even stopping idiopathic scoliosis.

## Conflict of Interest

The authors declare that there is no conflict of interest.
